# Recent Advances on Carbon Nanotubes and Graphene Reinforced Ceramics Nanocomposites

**DOI:** 10.3390/nano5010090

**Published:** 2015-01-20

**Authors:** Iftikhar Ahmad, Bahareh Yazdani, Yanqiu Zhu

**Affiliations:** 1 Center of Excellence for Research in Engineering Materials, Advanced Manufacturing Institute, King Saud University, Riyadh 11421, Saudi Arabia; E-Mail: ifahmad@ksu.edu.sa; 2 College of Engineering, Mathematics and Physical Sciences, University of Exeter, Exeter EX4 4QF, UK; E-Mail: by219@exeter.ac.uk

**Keywords:** nanocomposites, mechanical properties, interface, graphene, carbon nanotubes (CNTs), ceramics

## Abstract

Ceramics suffer the curse of extreme brittleness and demand new design philosophies and novel concepts of manufacturing to overcome such intrinsic drawbacks, in order to take advantage of most of their excellent properties. This has been one of the foremost challenges for ceramic material experts. Tailoring the ceramics structures at nanometre level has been a leading research frontier; whilst upgrading via reinforcing ceramic matrices with nanomaterials including the latest carbon nanotubes (CNTs) and graphene has now become an eminent practice for advanced applications. Most recently, several new strategies have indeed improved the properties of the ceramics/CNT nanocomposites, such as by tuning with dopants, new dispersions routes and modified sintering methods. The utilisation of graphene in ceramic nanocomposites, either as a solo reinforcement or as a hybrid with CNTs, is the newest development. This article will summarise the recent advances, key difficulties and potential applications of the ceramics nanocomposites reinforced with CNTs and graphene.

## 1. Introduction

Ceramics are potential contestants for diverse sophisticated engineering applications, and plenty of attentions have been focused to further improve their properties by adopting emerging technologies. As a result, much deeper understandings and significant amounts of improvements in their structures and properties have been achieved after decades of efforts. However, many challenge issues limit their wide applications, such as the degradation of high temperature mechanical properties of non-oxide silicon nitride (Si_3_N_4_) and silicon carbide (SiC), and low fracture toughness, poor creep, deprived thermal shock resistance of oxide ceramics like alumina (Al_2_O_3_) and zirconium oxide (ZrO_2_) [1]. To date, ceramics have found some niche applications, from high speed cutting tools, dental implants, chemical and electrical insulators, to wear resistance parts and various coatings, due to their high hardness, chemical inertness and high electrical and thermal insulating properties [2]. Low fracture toughness restricts ceramics for applications in aircraft engine parts and in extreme environments for space engineering [3]. Presence of impurities, pores and cracks cause pure ceramics extremely brittle, and complex/expensive processing technology is needed to reduce such fatal drawbacks. For decades, the addition of a second reinforcing phase in ceramics has been an effective practice to improve their toughness, converting brittle ceramics to practical engineering materials. Recent advances in nanomaterials have offered the opportunity to tailor the ceramic structures at nanometre scale, for the development of new classes of stronger, tougher engineering ceramics with added functionalities. Chosen nanomaterials with distinct morphologies and properties have been used to reinforce monolithic ceramics [4,5,6]. In particular, the exceptional mechanical behaviour and outstanding multifunctional features of carbon nanotubes (CNTs) and graphene have made them the wonder materials, standing out from many other nanomaterials, among different research communities. There has been much documented research attempting to incorporate both types of CNT in brittle ceramics to convert them into tough, strong, electric and thermal conductive materials [7,8,9,10,11,12,13,14,15]. Graphene, known as a monolayer of carbon atoms arranged in a honeycomb lattice, has shown similar properties to carbon nanotubes with impressive thermal, mechanical, and electrical properties, and is a promising alternative of CNTs in various applications [16,17,18]. Compared with CNTs, graphene also have large specific surface areas and they do not form agglomerates in a matrix when handled appropriately, thus an ideal nano-filler for composite materials. In this regard, the low-cost, high quality and commercially more viable a-few-layer-thick graphene nanosheets, designated as graphene nanoplatelets (GNPs) are more promising for practical engineering applications, thus attracted considerable research interests for advanced ceramic matrices. Indeed, various crucial ceramics such as Al_2_O_3_, Si_3_N_4_ and ZrO_2_ have been reinforced by the GNP fillers and obvious improvements in fracture toughness, thermal and electrical properties have been obtained [19,20,21,22,23,24,25,26]. However, research of ceramic-GNP nanocomposites is in its infancy, and more thorough and systematic studies are required.

Nevertheless, ceramics reinforced with CNTs, graphene and GNT (CNTs/graphene hybrid) have indeed showed significant improvements in the fracture toughness and other mechanical properties by following complex toughening mechanisms, although wide variations in the results still remain problematic. In fact, processing ceramic nanocomposites is complicated due to the introduction of a second reinforcement phase of nanometric scale. Conventional rules and benefits associated with microscopic reinforcement phases need to be modified carefully and validated fully before being applied directly. In this context, recent advances in the fabrication technology, mechanical properties and potential applications of typical ceramic nanocomposites reinforced with CNTs and graphene are presented in this paper. The main purpose of this review is to provide a comprehensive picture of the current state of research progresses and challenges concerning the graphene and CNTs-reinforced ceramic composites, to assist the ceramic community for further developments.

## 2. CNTs-Reinforced Ceramics Nanocomposites

### 2.1. Pre-Processing for Good Dispersion

**Figure 1:**
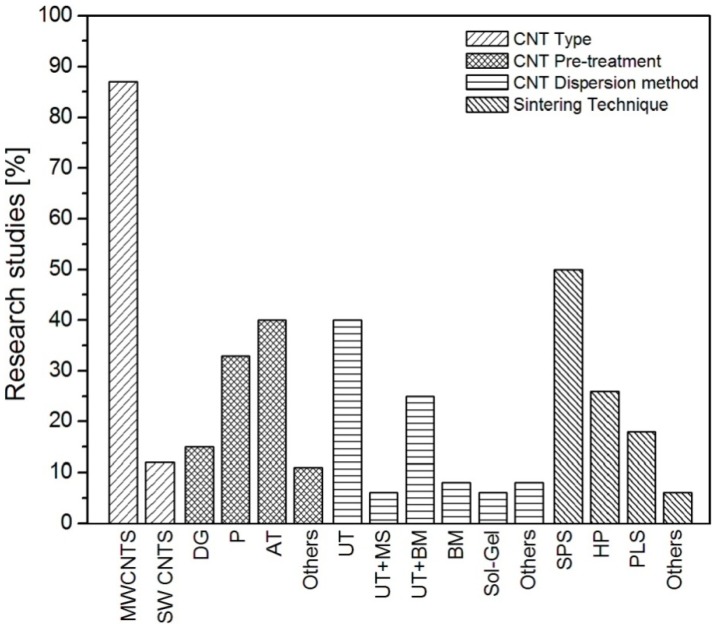
Statistical analysis of the carbon nanotubes (CNTs)-reinforced ceramic nanocomposites.

A statistical summary of the varieties of processes opted to fabricate CNTs containing ceramic nanocomposites is graphically presented in Figure 1, and Table 1 gives further details of these processes. It is evident that 88% of the reported cases used the readily available and economically feasible multi-walled carbon nanotubes (MWCNTs) as the reinforcement, against single-walled carbon nanotubes (SWCNTs). Nearly 40% adopted a wet oxidation process (treatment with concentrated H_2_SO_4_ and HNO_3_ in 3:1 ratio) to purify the CNTs in an effort to remove unwanted impurities including amorphous carbon nanofibres, carbon nanoparticles, amorphous carbon coating layers, and metallic catalyst residues; and about 33% of the reports attempted pristine CNTs; whilst the others tried oxidation through annealing [27,28,29,30]. The wet oxidation purification method for CNTs offers two folded advantages, realizing purification and simultaneous attachments of carboxyl functional groups onto the CNT surfaces which facilitates their mixing with and dispersions into matrices. It is a fact that CNT clusters prevent each individual CNT forming the ideal interconnection desired with the matrix, leading to ill-constructed interface and microstructures, detrimental to the final mechanical properties. Thus homogenous CNT dispersion within the matrix is extremely imperative. In addition, the actual quality of CNT dispersion is the foremost factor in tumbling the densities of CNT-reinforced ceramics, because homogenous dispersion is attainable only at low CNT concentrations (<2 wt% ), and higher than that normally ended up with severe agglomerations [31].

**Table 1:** Processing details of CNTs-reinforced ceramics nanocomposites.

Reference	Matrix	CNT types	Purification methods	Dispersion procedures	Sintering techniques
[10]	Si_3_N_4_	SW	P	UT of CNTs with surfactant (C16TAB) and Si_3_N_4_	SPS under vacuum
[12]	Al_2_O_3_	MW	Oxidation at 500 °C for 90 min	UT of CNTs in ethanol	SPS at 1500 °C for 10 min under 50 MPa
[32]	Al_2_O_3_	MW	AT (H_2_SO_4_ + HNO_3_)	UT of CNTs into water and SDS then incubation for 2 weeks	HP at 1600 °C, 60 min, 40 MPa
[33]	Al_2_O_3_	MW	AT (H_2_SO_4_ + HNO_3_) for 3 h	24 h BM of ball Al_2_O_3_ powder and 30 min UT of CNTs in water and then BM of CNTs/Al_2_O_3_ mixture	PLS at 1500–1600 °C, 120–240 min, Ar
[34]	Al_2_O_3_	MW	Pristine	UT of CNTs for 1 h in alcohol	CIP at150MPa and PLS at 1500 °C, and 1700 °C with 2 h
[35]	Al_2_O_3_	MW	AT (heating in 65% HNO_3_ at 80 °C for 8 h)	BM and Surfactant (Darvan C–N)	PLS at 1500 °C for 2 h using Ar
[36]	Mulite	MW	P	CNTs dispersion into ethanol by MS and UT	HP at 1600 °C for 60 min under Ar atmosphere at 30 MPa
[37]	Si_3_N_4_	MW	P	24 h ball milling the CNTs and Si_3_N_4_ slurry	HP at 1750 °C for 60 min under 30 MPa
[38]	ZrB_2_–SiC	MW	P	20 min UT of CNTs and matrix with subsequent 24 h ball milling	HP at 1900 °C for 60 min under 30 MPa
[39]	BaTiO_3_	MW	P	-	HP, 1200 °C, 60 min
[40]	Al_2_O_3_	MW	-	DG (CVD at 750 °C for 15 min for direct CNTs growth on Al_2_O_3_ nano-particles)	SPS at 1150 °C for 10 min under 100 MPa
[41]	Al_2_O_3_	SW	Pristine	UT of CNTs in ethanol	SPS at 1520 °C under 80 MPa
[42]	Al_2_O_3_	MW	P	35 h UT in water	SPS at 1300 °C, 20 min, 90 MPa
[43]	Al_2_O_3_	MW	AT	UT of CNTs and Al_2_O_3_ in water followed by 2 h and BM of CNTs/Al_2_O_3_	PLS at 1600 °C, 15 min, Ar
[44]	Al_2_O_3_	MW	AT (HNO_3_ for 30 min)	5 h BM of CNTs and 1 h UT of CNTs. 5 h BM of CNT/Al_2_O_3_ in ethanol	PLS at 1550 °C, Ar
[45]	Al_2_O_3_	MW	AT (H_2_SO_4_ + HNO_3_ in 3:1 for 7 h)	surfactant (SDS) using combination of UT and 24 h BM	HP at 1550 °C for 1 h under 30 MPa using Ar gas
[46]	Al_2_O_3_ + ZrO	MW	AT (heating in 65% HNO3 at 80 °C for 8 h))	2 min UT of CNTs with surfactant (SDS)and 24 BM then freezing with Nitrogen	HP at 1500 °C for 2 h under 30 MPa in Ar atmosphere
[47]	Al_2_O_3_	SW	AT (H_2_SO_4_ + HNO_3_)	UT for 24 h	SPS at 1300 °C for 5 min under 75 MPa

Notes: SW: Single-walled CNTs; MW: Multi-walled CNTs; UT: Ultrasonication; BM: Ball milling; HP: Hot-pressing; SPS: Spark plasma sintering; PLS: Pressureless sintering; SDS: Sodium dodecyle sulphate; CIP: cold isostatic pressing; P: Pristine; MS: Magnetic stirring.

To combat this dispersion issue, as shown in Figure 1, the most dominant (40%) technique involving colloidal technology (ultra-sonication of CNTs for different durations into different solvents with or without surfactants). Until recently, attempts increasingly focused on a combined process (colloidal technique and ball milling) which have produced better results and was more reproducible than other techniques (ball milling, sol-gel, planetary centrifuge mixing, magnetic stirring, tape casting, *etc.*), as shown in Table 1. Moreover cationic, anionic and neutral surfactants have greatly contributed to the detangling of CNT clusters, of which SDS (sodium dodcyle sulphate) seems to be the most used one [11]. In addition, several reports described the growth of CNTs directly onto the surface of ceramics nanoparticles using a standard chemical vapour deposition (CVD) technique; however, this method failed to create high quality coverage, on top of the low yield issues [13].

Cultivation of CNTs in porous ceramics is intriguing process, and numerous efforts have been documented for highly ordered CNT growth within the pores of thin SiO_2_ and Al_2_O_3_ membranes, which led to novel CNTs-reinforced porous ceramics with potential applications as field emitters, nanocapacitors, and scanning microscope probes [48,49,50,51,52]. To prepare CNTs carrying porous ceramic composites, Fan *et al.* [27] first embedded catalyst inside the SiO_2_ pores of the ceramics pores, then allowed for the carbon source to diffuse into and deposit inside the pores; whilst Kyotani *et al.* [48] grew CNTs on an anodic aluminium oxide (AAO) porous membrane with and without catalyst. The CNT growth mechanism in a catalyst free CNTs-porous ceramic is still not completely understood. The AAO membrane template may itself catalysed the CNT growth by deposition of carbon atoms on the internal pore surface of the complex three dimensional structure, as proposed by Sui *et al.* [49]. Since catalyst facilities carbon source decomposition, thus further deposition of atomic carbon tends to result in more ordered or well-crystallised structures, leading to better quality nanocomposites than the non-catalysed process. Patterning and lithography technology enabled Bae *et al.* to deposit a thin Si layer on an AAO substrate for enhanced CNT growth [50]. Parham *et al.* have recently prepared a 3 wt% CNT-containing composite using Al_2_O_3_ and SiO_2_ porous ceramics, and resulting composites exhibited a high efficiency for yeast cell filtration (98%), a 100% heavy metal ions removal from water and excellent particulate filtration performance from air [51,52].

Despite these achievements for CNT dispersion in various ceramic matrices, some known issues still remain. For example, SWCNTs are always produced in the form of bundles of tens of nanotubes, and their separation into individual tubes is still extremely difficult, concerning the nanocomposite fabrication. This area thus needs further investigations, because SWCNTs have promising applications in biomedical engineering, composite technology and nanodevices [30]. Dispersion of CNTs within ceramic matrices is generally assessed by the microscopic images of the fractured surfaces of nanocomposite samples taken from desired areas of interest, and the representativeness of this method sometimes is a concern, as it may not be a true reflection of the CNT dispersions for other locations [53,54,55]. Therefore, the standardization of CNT dispersion assessments in composites beyond ceramics is vital for quality control in manufacturing and industrial applications.

### 2.2. Densification Processes

**Figure 2:**
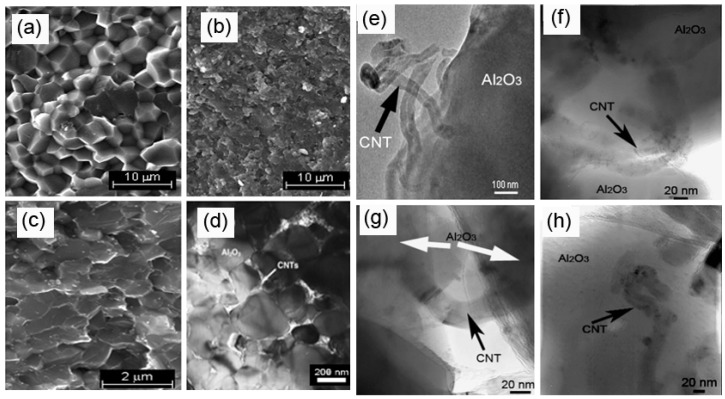
Structural features of (**a**) Monolithic Al_2_O_3_ showing large grains with inter-granular fracture; (**b**) CNTs/Al_2_O_3_ nanocomposites with fine grains; (**c**) Trans-granular fracture mode in CNTs/Al_2_O_3_ nanocomposites; and (**d**) Single-walled (SW)CNTs at grain boundary of Al_2_O_3_ matrix. TEM images exhibiting the CNT–ceramic interactions (**e**) Multi-walled (MW)CNTs (black arrow) showing their morphology in nanocomposite; (**f**) A single MWCNT existing at grain boundary; (**g**) in porosity and (**h**) Embedded within a single ceramic grain. Adapted from References [32] and [66] with permissions. Copyright 2010, Elsevier Ltd.

Achieving near full density, without damaging the CNT structure and morphology, is a fundamental requirement and another important challenge in ceramic matrix nanocomposite technology, as most of the mechanical properties are strongly affected by the density. CNTs hinder the ceramic grains coalescence by existing at the grain boundaries, which tends to lead to poorly densified microstructures [56]. For this reason, pressure-assisted consolidification processes are generally be used to counter this problem. Figure 1 shows that about 76% nanocomposites were consolidated by pressure-assisted sintering processes, in which spark plasma sintering (SPS) and hot-pressing (HP) have a share of 50% and 26%, respectively. Hot-pressing provides simultaneous high pressures and high temperatures to powder systems, which in turn gives high densities, thus good mechanical properties to either pure ceramics and their composites. Coble *et al.* explained that the enhanced densities were associated with accelerate densification due to higher stresses caused by external pressure, and this phenomenon consolidated the grains to a desirable density; unfortunately, damage to the CNTs during the matrix grain growth could occur due to prolong sintering at extremely high temperatures which was a potential big shortcoming of HP [32,57]. The structural damage problem of CNTs during HP can be avoided by using SPS technique, in which near full densification is achievable at lower sintering temperatures with substantially short holding time. The microscopic images (Figure 2) showed that the CNTs were mainly located at the ceramic grain boundaries, well adhered with the matrix without apparent damage to the structure and morphology [58]. Pressureless sintering (PLS) offers convenient and cheaper consolidation alternations, but wide variation in earlier results have made this technique unattractive and debatable. For example, Zhan *et al.* [15] and Ahmad *et al.* [59] claimed widely different densities for similar samples, as high as 99% and as low as <90% for 1 wt% CNTs-reinforced Al_2_O_3_, respectively. However in recent reports, Sarkar *et al.* [34] densified Al_2_O_3_ containing 0.3 vol% of MWCNTs to >99% at 1700 °C using PLS sintering; and similarly Michalek *et al.* [35] and Ghobadi *et al.* [60] obtained 99.9% and >98% densities for Al_2_O_3_ reinforced with 0.1 wt% and 1 vol% MWCNTs, respectively. Regarding other ceramics, Tatami *et al.* [61] achieved Si_3_N_4_-MWCNTs composites by pressureless sintering during which the constituents including sintering additives were initially pressed uniaxially and subsequently sintered in furnace at 1700 °C under N_2_ atmosphere. They recorded a drop in relative densities from 100% to 90% for 0 to 5 wt% CNT additions; whilst they obtained much higher densities >96% for 5 wt% MWCNT additions by using HP. Microwave sintering is another inspiring and “green” sintering technique, with the advantage of lower densification temperatures and shortened processing time. This technique has been successfully used to consolidate most mainstream industrial ceramics (e.g., Al_2_O_3_, ZrO_2_, Si_3_N_4_), both pure and composite forms, and resulted in high densities, due to rapid microwaves heating characteristics [62,63]. Nevertheless, the mixed large and fine grained microstructure of the final ceramic consolidated by microwave sintering has made it bit divisive, but this technique exhibits great potentials for CNTs-reinforced ceramics’ densification. The advantageous features such as short sintering time and low densification temperatures are not deleterious for CNT structures; furthermore the localize heating at the grain boundaries may be helpful in constructing strong interfaces between CNTs and the ceramic matrices; finally, the grain coarsening seems not a big issue in microwave sintered CNTs-reinforced ceramics, possibly owing to the grain refining tendency of CNTs [64,65]. Table 1 covers the HP, PLS and SPS these main methods.

In seeking of highly dense composite structure, new techniques are always attempted, and we will summarise a few diverse and interesting methods here. For example, in order to protect the CNT structures by preventing reactions with SiC during high temperature integration, Thostenson *et al.* first prepared a preform of SiC nanoparticles and CNTs (duly dispersed in polymer matrix) then carbonized the perform followed by infiltrating molten Si into the preform under vacuum at 1400 °C to claim dense nanocomposites; whilst Wang *et al.* packed SiC nanoparticles and CNTs in a cylinder and arranged graphite heater inside the cylinder followed by heating up to 1700 °C to consolidate the CNTs-SiC mixture [36,67,68,69].

### 2.3. Microstructural Analysis

Sharp reduction from coarser grains in monolithic ceramics (Figure 2a) to finer grains in CNTs-reinforced ceramics (Figure 2b) is a principle feature of structural change, occurred due to the pinning of matrix grains by CNTs which restricted the grain growth during sintering [66]. Fracture mode alteration from inter-granular in monolithic ceramics (Figure 2a) to trans-granular in the CNTs-reinforced ceramics (Figure 2c) is another interesting change being revealed. The morphological analyses of fractured surfaces are helpful in depicting the mechanisms behind such changes. In the case of monolithic Al_2_O_3_, it shows clearly the edge and corner fractural features (Figure 2a), representing the typical inter-granular fracture mode; and conversely a blurry and glaze-like surface appears for CNTs-reinforced Al_2_O_3_ (Figure 2c), indicating the trans-granular mode of fracture [66]. These observations mean that CNTs, as the second phase, must be responsible for altering the fracture modes. Indeed, when CNTs were homogenously dispersed within the ceramic matrix, they arranged themselves at various locations such as along grain boundaries (Figure 2f), across grains boundaries (Figure 2g), inside single grains (Figure 2h), contributing to strengthening the composites at nanometre level by making bridges across grains and sharing the grains, as discussed in prior studies [66,70]. Presumably, all these interesting arrangements of CNTs in ceramic matrices promoted the trans-granular fracture, rather than inter-granular fracture as did in the pure ceramic. In a very recent report, Ahmad *et al.* obtained 5-fold finer grain size in MWCNTs/Al_2_O_3_ nanocomposites by 300 ppm Y_2_O_3_ doping than its undoped Al_2_O_3_ counterpart, and mixed inter/intra fracture mode in Y_2_O_3_ doped nanocomposites was observed [31,37]. However this fracture mode change phenomenon is another grey area that is not fully understood for CNTs-reinforced ceramics, which offers opportunities for prospective thinking and further research work.

### 2.4. Mechanical and Functional Properties

In view of the vast applications of the economically viable Al_2_O_3_ ceramics in industry, lots of studies have been done to improve their fracture toughness by CNT additions. However, inconsistent results (Table 2) put question marks on these triumphs and core issues in such discrepancies were found in the CNT dispersion methods, choice of sintering process and techniques adopted for characterisation [34,40,41,50,59,61,71,72]. For example, Table 2 shows that the higher fracture toughness values of MWCNTs-reinforced Al_2_O_3_ were obtained at lower CNT additions (<2 wt%), and declining trend can be seen at higher CNT levels in all cases, except from the values reported by Zhan *et al.* [15]. Furthermore, the composite fracture toughness reported by Zhan *et al.* [15] was the highest in Table 2. However, this value may be due to various factors: (1) the use of SPS techniques (positive); (2) reinforcement phase being SWCNTs (positive); and (3) the assessment of the fracture toughness by an unreliable direct crack method, DCM, (negative). Yamamoto *et al.* [12] used the SPS to sinter similar composite reinforced with MWCNTs, and used the single-edged notched beam (SENB) method to assess the fractures toughness, however the results were now as good as the results reported by Zhan *et al.* [15] and Ahmad *et al.* [31]. In case of the high values reported by Ahmad *et al.* [66], it is probably due to the better dispersion of CNTs within the matrix, as they adopted a unique method. Further, Huang *et al.* [39] showed tremendous improvements in fracture toughness (57%, 114% and 328%) values for BaTiO_3_ ceramic after reinforced with (0.5, 1 and 3 wt%) MWCNTs; whereas a 15% improvement was recorded by Tian *et al.* [38] for 2 wt% MWCNTs-reinforced ZrB_2_-SiC ceramics.

**Table 2:** Properties of CNTs-reinforced ceramics.

Reference	Matrix	CNT contents	Relative density (%)	Hardness (GPa)	Flexural strength (MPa)	Fracture toughness (MPa. m^1/2^)
[10]	Si_3_N_4_	0	99.2	15.7	1046	4.8
		1 wt%MWCNTs	98.7	15.0	996	6.6
[12]	Al_2_O_3_	0	95.6	17.3	500	4.4
		0.5 wt% MWCNTs	99.2	16.8	685	5.9
		1 wt% MWCNTs	98.9	15.9	650	5.7
[15]	Al_2_O_3_	0	-	-	-	3.3
		3 wt% SWCNTs	-	-	-	7.9
[27]	Al_2_O_3_	0	97.7	-	326	3.08
		6 wt% MWCNTs	95.4	-	314	5.55
[32]	Al_2_O_3_	0	99.8	16	356	3.5
		2 wt% MWCNTs	99.5	18	402	6.8
		5 wt% MWCNTs	99.1	-	423	5.7
[34]	Al_2_O_3_	0	99.5	17.5	222	3.92
		0.15 vol% MWCNTs	98.4	21.4	242	5.27
[35]	Al_2_O_3_	0	-	16.9	-	5.5
		1 vol% MWCNTs	-	13.5	-	6.0
[36]	Mulite (3Al_2_O_3_ + 2SiO_2_)	0	-	-	466	2.0
		2 wt% MWCNTs	-	-	512	3.3
[69]	SiC	0	939	-	303	3.3
		10 wt% MWCNTs	94.7	-	321	3.8
[38]	ZrB_2_-SiC	0	-	15.8	582	4
		2 wt% MWCNTs	-	15.5	616	4.6
[39]	BaTiO_3_	0	98.5	-	-	0.7
		98.50	98.5			0.7
		0.5 wt% MWCNTs	97.3			1.1
		1 wt% MWCNTs	99.2			1.5
		3 wt% MWCNTs	98.6			3.0
[73]	Al_2_O_3_	0	-	-	395	4.41
		20 vol% MWCNTs	-	-	403	4.62
[74]	Al_2_O_3_	0	-	-	-	3
		1 wt% MWCNTs	-	-	-	5
[75]	Al_2_O_3_	0	-	15.71	-	3.24
		5 wt% MWCNTs	-	0.72	-	4.14
[76]	Al_2_O_3_	0	-	18.2	-	4.5
		2.5 wt% MWCNTs	-	15.75	-	11.4
[77]	Al_2_O_3_	0	99.9	22.9	-	3.54
		10 vol% MWCNTs	97.4	11	-	2.76

Recently, Sarkar *et al.* [34] calculated fracture toughness values of the Al_2_O_3_–MWCNT nanocomposites by employing DCM method using Niihara and Liang models, and reported better fracture toughness values than those obtained using SENB technique; whereas Ahmad *et al.* [66], reported higher fracture toughness values attained from SENB method than those obtained from DCM method using Chantikul model. These conflicting reports suggest that engineering components cannot be validated for structural load-bearing applications using DCM method; however, this convenient method is widely employed for fracture toughness comparisons [31]. Similar inconclusive and controversial fracture toughness values regarding CNTs-reinforced Si_3_N_4_ were also reported by Corral *et al.* [10] and Pasupuleti *et al.* [37] Both consolidate Si_3_N_4_ with 1 wt% CNTs and obtained 30% reduction (by SENB method) and 40% increment (by ISB method) in fracture toughness, respectively.

Regarding other mechanical properties such as hardness and elastic modulus, Yamamoto *et al.* [12] investigated a range of MWCNT additions in Al_2_O_3_ ceramics and concluded a drop in hardness and rise in flexural strengths at low MWCNT additions and further reduction in both properties by adding more MWCNTs, and consistent results were reported by many others for the same material system, as shown in Table 2. Pasupuleti *et al.* [37] showed a small decrease in hardness (4%) and flexural strength (5%) for 1 wt% MWCNTs-reinforced Si_3_N_4_, however, Corral *et al.* [10] reported a much severer 45% reduction in hardness for same reinforcement contents in Si_3_N_4_.

The dual role of CNTs, indirectly enhancing the mechanical properties and directly acting as lubricant, converts ceramic composites into an attractive wear resistance material, and various reports demonstrated the steady reduction of friction coefficient with CNT additions [78]. High thermal and electrical properties of the CNTs have been predicted and several attempted to incorporate CNTs into insulated ceramics in order convert them into highly electrical and thermally conductive materials [73]. Ceramics exhibited higher electric conductivity (EC) when reinforced with SWCNTs (10^6^ S/m) than with MWCNTs (10^3^–10^5^ S/m) [40,79]. Sarkar *et al.* reported that the EC of MWCNTs-reinforced composites was dependent on the formation of electrically conductive networks by dispersing the CNTs homogenously in the matrix, and on the grain sizes of the final nanocomposites, as larger grain size with less grain boundaries showed better results [80]. So far Estili *et al.* has obtained the highest EC of 4816 S/m for Al_2_O_3_-20 vol% MWCNTs, which is 43% higher than that reported by Zhan *et al.* [73,79]. In addition, Kumari *et al.* obtained an exceptional value of 3336 S/m by reinforcing Al_2_O_3_ with 19 wt% MWCNTs nanocomposites, however, at the cost of poor mechanical properties [41]. In contrast to MWCNTs, the SWCNTs reinforcement into the Al_2_O_3_ matrix offered better conductivity of 3345 S/m without compromising mechanical properties [71]. Zaman *et al.* studied the effects of surface functionalization of the SWCNTs on EC and reported that the hydroxyl group functionalized SWCNT offered ~10 times higher EC in 1 wt% SWCNTs-reinforced Al_2_O_3_ nanocomposites than those functionalized by carboxylic acid group [72]. Moreover, Bi *et al.* reported a drop in the electrical percolation by increasing the aspect ratios of MWCNTs [81]. Although the thermal conductivity (TC) of SWCNTs and MWCNTs are ranges from 3000 to 6000 W/m·K, however, their nanocomposite with ceramics barely demonstrated good thermal performance. Compared to unreinforced Al_2_O_3_, Zhan *et al.* reported lower (7.3 W/m·K) TC in nanocomposites reinforced with 15 vol% SWCNTs than their monolithic counterpart (27.3 W/m·K) [71]. Both Kumari *et al.* and Bakshi *et al.* reported higher TC values (63.52 and 6 W/m·K) in nanocomposites with (8 and 4 wt%) MWCNTs than those of pure Al_2_O_3_ (19.96 and 5.37 W/m·K) samples, respectively [82]. This area of research is therefore more complicated and interesting.

### 2.5. CNTs/Ceramic Interface and the Toughening Mechanism

Reinforcement (fibres or whiskers) pullout is the main toughening mechanism in conventional ceramics, which is further associated with weak interfacial connection of reinforcement with matrix. Same classical approach was proposed for toughness mechanism in CNTs-reinforced ceramics in several initial reports [83,84,85]. However, in later research Padture *et al.* and many others observed that micro-structural features of CNTs-ceramics were immensely dissimilar from conventional composites, and these observations strongly suggested that existing microscale mechanism may not be fully applicable to CNTs–ceramic systems [18,23,61]. Microstructure of conventional ceramic composites consists of inflexible and straight reinforcement, and the interface is optimally designed in such a way that it debonds on applied load [86]. Imagine, when a reinforcement encounters a crack then it bridges the crack in its wake, pullout does frictional work and these together effectively make crack propagation more difficult, in addition to this large reinforcement dimensions lead to longer crack-wake bridging zones and consequently resulted in higher toughness [85,87]. Meanwhile, these large reinforcements prompt larger flaws and turn strength to lower values. In contrast, CNTs are highly flexible, hollow nanometre sized fibres, therefore the toughening mechanism may be entirely different from conventional ones. Frictional pullout of fibres occurred in classical composites may not be the only toughening mechanism in CNTs-reinforced ceramics. Accordingly, new concepts and philosophies of uncoiling and elastic stretching of CNTs during the crack propagation were proposed as main toughness mechanisms by Padture *et al.* [7]. During crack propagation, initial uncoiling of CNTs occurs in the crack wake, and when the crack further propagates the uncoiled CNT stretches elastically serving as stretched CNT bridges instead of conventional frictional pull-out bridges, thus impedes the crack propagation, as shown in Figure 2g [66]. These concepts are convincingly identified the role of CNTs as an individual entity, also applied to the cluster form. Surface damages to SWCNTs during purification and subsequent sintering process are well-known, and in this picture the role of CNT’s elastic stretching in toughness is slightly litigious. In this regard, mathematical modelling will be a helpful tool in explaining the role of CNTs in strengthening ceramics and predicting their behaviour in services.

Recent developments in the electron microscope technology are changing the research approaches, and attentions are now tending to focus on tailoring the interface structure at atomic level, to construct defect-free structures with interesting functionalities. FIB-SEM (focused ion beam scanning electron microscopy) has made the scientists’ life not as hard as ages ago. A TEM (transmission electron microscope) sample of hard materials, e.g., ceramics can be prepared in hours, which was a laborious task to arrange for days and even weeks earlier. For CNTs-Al_2_O_3_ nanocomposites, due to the interesting reaction of Al_2_O_3_ with alkaline, a simple powder etching process can always be used to collect CNTs with a thin layer of Al_2_O_3_ residue, for interface study under TEM. Similar results are obtained when compared with FIB-SEM [66].

**Figure 3:**
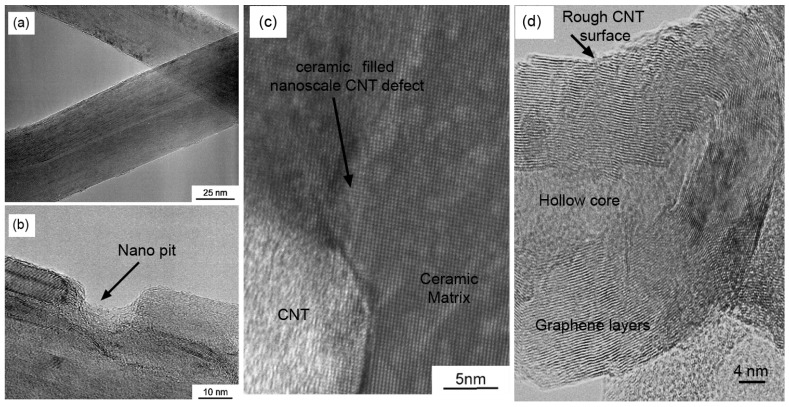
(**a**) TEM image of the pristine MWCNTs; (**b**) High-magnification TEM image of the acid-treated MWCNT surface, arrow indicates nano-pit; (**c**) Nano-pit on the acid-treated MWCNTs is filled up with Al_2_O_3_ crystal; and (**d**) Rough surface of MWCNT produced by chemical vapour deposition (CVD) method. Adapted from References [12] and [32] with permissions. Copyright 2009, Elsevier Ltd. and 2008 IOP Publishing Ltd.

Back to CNTs-reinforced ceramics, where the interface controls the CNT debonding, pullout and crack-bridging at micron and nanometre level, these different mechanisms act as energy dissipative processes during mechanical loading. Physically, an interface is a complex transitional region layered between the reinforcement and parent matrix thus, the control of interface chemistry and tailoring smart microstructures are essential steps for producing exceptionally tough and strong nanocomposites. Dedicated efforts have been done to explore the CNTs-ceramic transition region and each addressed in interesting way [12,66]. Indeed, rough surface of CNTs produces the required frictional forces which resist in detaching CNTs from the ceramic matrix. Yamamoto *et al.* proposed that acid treatment does not significantly damage the overall structures of MWCNTs however, localized etches of the cylindrical body at different locations create nanoscale defects (nano-pits) along the tube axis, as shown in Figure 3b [12]. These nano-pits having depths of ~15 nm are anchored by the matrix grains (Figure 3c), forming locks and resistance in MWCNTs’ sliding over the matrix, thus leading to good connection of composite constituents at the interface [12]. Further, a close cross-sectional look of the MWCNT shown in Figure 3d of the high resolution TEM image reveals its uneven surface, hollow core and graphitic layers. These layers are not concentrical on a long distance and many compartments exist, which is a typical feature of MWCNTs synthesized by CVD. Ahmad *et al.* [78] postulated that high surface roughness of the CNTs could result in two potential advantages like chemically highly reactive and physically difficult to slide out of the matrix, compared with a smooth surface. The former could help to improve the interfacial bonding with the matrix and the latter can pose much larger friction forces to stop the CNT pullout [78].

**Figure 4:**
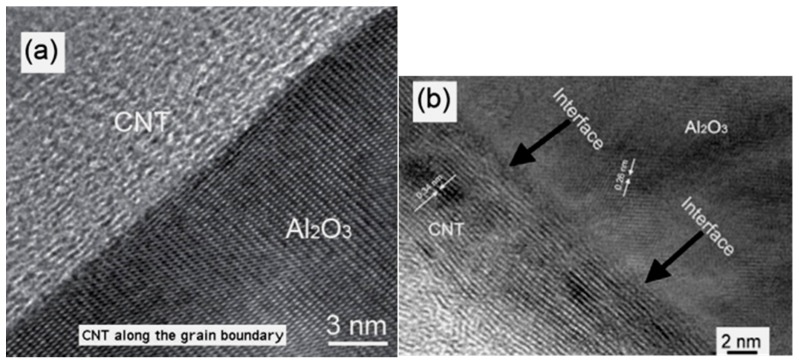
(**a**,**b**) High-resolution TEM images showing CNT/ceramic interfaces. Adapted from References [15] and [32] with permissions. Copyright 2005 Advanced Study Center Co. Ltd. and 2010 Elsevier Ltd.

The CNT’s surface unevenness and its anchoring with the ceramics matrix are a good physical explanation of enhanced frictional forces at the interface. However, the chemical interactions of CNTs with the ceramics remained unattended for several years. Estili *et al.* [88] rigorously studied the interfacial areas of CNTs/Al_2_O_3_ nanocomposites using high resolution-TEM, but unable to identify any interfacial phases or intermediate compounds at the CNTs/Al_2_O_3_ interface. A recent attempt addressed this topic and explained the chemical activity took place at the CNTs/Al_2_O_3_ interface during HP process, and reported the formation of an extremely thin (1–2 nm) intermediate phase of Al_2_OC, which is possibly produced due to the carbothermal reduction of Al_2_O_3_ by CNTs [78]. Figure 4a–b show clear evidence of a CNT sticking with Al_2_O_3_ at the interface.

Owning to the multi-layer graphene structure of MWCNTs, the possibility of such chemical and physical reactions with the accommodation of nano-pits and eating of few outer layers for Al_2_OC or Al_4_C_3_ formation can be justified. However, this may not be true for SWCNTs which contain only a single graphene layer while forming the tubular structure, even plenty of studies claimed tremendous improvements in ceramics properties [22,26]. This raises one big question as to being only one layer how it reacts with the matrix to form a good interface following the toughening mechanisms proposed above. Therefore, this mystery remains unresolved. The understanding of the nanostructure characteristics and the interfacial relationship between SWCNTs and the ceramic matrices is far from satisfactory, which opens new windows of potential research in this advanced area of nanotechnology [18,26,40,61].

## 3. Graphene Reinforced Ceramic Nanocomposites

### 3.1. Raw Materials

As a cousin of CNTs, the 2D graphene bears many similarities to CNTs in terms of nanocomposites application. For bulk engineering nanocomposite applications which require large volume amount, a few layered graphene platelets or flakes, including the reduced graphene oxide (GO), are far more viable and economical than single layered graphene. Therefore, the term graphene in this context refers to graphene nanoplatelets (GNPs).

In composite applications, both the mechanical exfoliation and reduction from GO have been used successfully [89,90,91,92,93,94]. In the mechanical cleavage method, commercial graphite powder (Aldrich) has been milled intensively in high efficient attritor mill in the presence of ethanol for 10 h, then the produced GNPs were mixed with ceramic powder [89]. The second method uses the Hummers’ process to produce GO, then using this water soluble GO to mix with ceramic powders [90]. In general, both mechanical milling and hummers method suffers from various sizes and thicknesses for the former due to lack of control on the milling energy and from surface structural damage for the latter originated from the oxidation [91], which will have negative effects on the final properties of the ceramic composites. Therefore, better quality control of the GNPs is of fundamentally importance for high quality nanocomposites development.

### 3.2. GNS Dispersions Processes

As discussed above for CNTs, mixing step is an equally challenging step in preparing graphene-reinforced ceramics composites. To avoid any damage and reduce agglomeration of GNPs will help to achieve high mechanical and physical properties. In essence, the dispersion of GNPs in fact is easier than CNTs, as the difficulties accompanied in CNT’s dispersion such as high aspect ratio and van der Waals interactions which cause CNT bundling are absence for GNPs. In addition, high specific area and 2D geometry of GNPs offer better disperseability in ceramic matrices. As a younger cousin to CNTs, the gained knowledge for CNTs can generally be used as a reference for GNPs-reinforced ceramic composites. Thus in this context, focus will be mainly on the different features with comparison.

Wet powder mixing are successful to disperse CNTs in ceramic matrixes [92,93,94,95], whilst for GNPs the choice of solvents are much wider than processing CNTs. Isopropyl alcohol NMP, DMF have all be used to mix with various ceramic matrices such as Al_2_O_3_, Si_3_N_4_, and ZrO_2_ powders. This drawback of this technique is energy consuming, and might cause damage to the GNP reinforcements. Colloid processing is a modified wet mixing process, and the key is to produce stabilized suspensions from GNTs and ceramic particles by changing their surface chemistry which facilitates homogeneous dispersion of GNPs. Anionic or cationic surfactants are generally used to alter the surface charge of GNPs, to positive or negative respectively, followed by adding them to a ceramic suspension with the same/opposite charges to form an homogenous ceramic-GNP dispersion. This hetero-coagulation process is a very effective route for well-dispersed ceramic composites [12,91]. Starting with GO, Wang *et al.* [20] used such electrostatic attractions between GO and Al_2_O_3_ particles to obtain homogenous dispersions of GO in Al_2_O_3_ powder first, followed by subsequent reduction of GO, who achieved a 53% and 13 orders of magnitude improvement in fracture toughness and conductivity. Walker *et al.* [25] used CTAB in both the GNPs and Si_3_N_4_ suspensions for mixing, and resulted in a 235% improvement in fracture toughness with only 1.5 vol% GNP addition.

### 3.3. Sintering Techniques

The densification of GNPs-reinforced ceramic nanocomposites also includes pressureless sintering, HP, SPS and HIP (hot-isostic pressing). The low temperature requirement and fast sintering rate advantages of SPS made it widely used for ceramic nanocomposites filled with carbon nano-fillers [20,24,43,95,96,97,98]. However, there are a few groups reported very good GNPs-reinforced ceramic nanocomposites based on HP densification [97,98,99,100]. For example, the GNPs-Si_3_N_4_ nanocomposites reported by Rutkowski *et al.* [99] showed improved thermal properties. After comparing the HP and SPS processes for GNPs-Al_2_O_3_ nanocomposites, Inam *et al.* [98] found out that the structural integrity of graphene from HP process is better than SPSed samples, with higher crystallinity, thermal stability and electrical conductivity, and was attributed to the thermally induced graphitization caused by longer sintering condition in a HP.

### 3.4. Structural Features, Mechanical Properties and Toughening Mechanisms

Toughening ceramic is one of the main research objectives for GNP nanocomposites, whist other benefits such as flexural strength and hardness can also be obtained. Using only 1.5 vol% the flexible 2D GNPs as a reinforcement for Si_3_N_4_, Walker *et al.* reported a 235% improvement in the toughness, and found GNPs anchoring with or wrapping around Si_3_N_4_ grains [25], thus blocking the crack propagation through the GNPs. This is the first time that such toughening mechanism was observed, and is a major different from the 1D CNTs. The same effective anchoring toughening was also confirmed by Liu *et al.* [24] in their GNPs/Al_2_O_3_ system, documented a 30.75% increase in flexural strength and a 27.20% increase in fracture toughness. These securely anchored GNPs around Al_2_O_3_ grains can form large area of interfaces with the matrix, increasing the interfacial friction, therefore the energy required for pulling out GNPs from the matrix will be greater than pulling out CNTs. They also successfully extended their process to a GNPs-reinforcing the ZrO_2_-Al_2_O_3_ system using SPS [24], in comparison with CNTs. The authors believed that due to similar mechanical properties to CNTs, and better dispersability GNPs are an effective alternative for CNTs in ceramic composites. In Si_3_N_4_ matrix, Tapaszto *et al.* [100] showed that GNPs indeed outperformed CNTs. However, it should be noted that, due to the larger contact area between GNPs and the matrix grains, the interface quality plays a more important role in toughening the ceramics than CNTs and other reinforcement phases.

**Figure 5:**
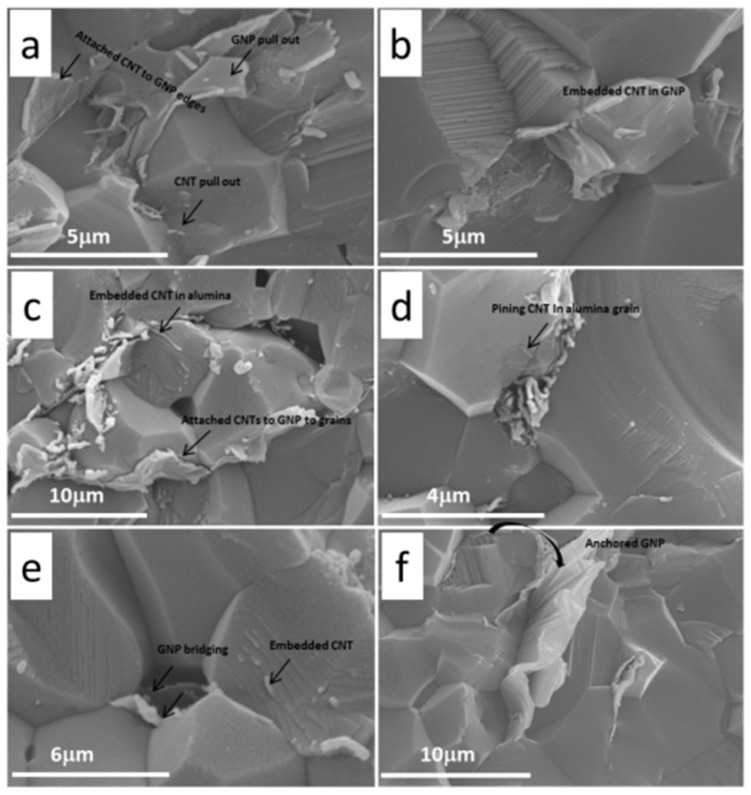
SEM images from fractured surface of GNT-Al_2_O_3_ nanocomposites with various GNP/CNT ratio, (**a** and **b**); Al_2_O_3_-(0.5 wt% GNP + 1 wt% CNT), (**c–e**); Al_2_O_3_-(0.5 wt% GNP + 0.5 wt% CNT), (**f**); Al_2_O_3_-0.5 wt% GNP. Adapted from Reference [26] with permission. Copyright 2014 Elsevier Ltd.

The different roles of CNTs and GNPs separately in ceramic matrices were well-documented, however within the same matrix could be more complex. A combination of the various advantages of different reinforcement phases, the very nature of the composite concept, could lead to superior properties, however this has rarely been investigated so far in ceramics. Very recently, Yazdani *et al.* [26] reported both 63% and 17% improvements in fracture toughness and flexural strength by using such a hybrid (MWCNTs + GNPs = GNTs) reinforcement phase in Al_2_O_3_ matrix. In their report, the role of GNPs and CNTs has been investigated separately. As evident in Figure 5, a large GNP rolled around Al_2_O_3_ grain due to its flexibility and produced large area of interface with Al_2_O_3_ matrix; therefore, it increased the required pull out energy during fracture and strengthened the grain boundaries so the fracture occurred through the Al_2_O_3_ grains rather than along the grain boundaries, whist MWCNTs contributed more to the bridging effect due to their higher aspect ratio. It is believed that MWCNTs can be stretched much longer than GNPs before collapsing during crack propagation. These roles are complementary with each other at appropriate concentrations, allowing for absorbing more energy during crack propagations.

## 4. Potential Applications

Owing to the improved fracture toughness and ancillary benefits of electrical and thermal properties, ceramics reinforced with CNTs and GNPs are promising for numerous prospective applications in the field of photonics, biomedical, automotive and aerospace engineering. Firstly, associated with the enhanced mechanical performance of Al_2_O_3_, the significantly improved wear resistance property of these composites could be suitable for a number of wear and sliding applications in automobile industry like cylinder lines, valve seat and piston rings [101]. Secondly, the SiC, Si_3_N_4_ and BaTiO_3_ systems filled with CNTs made them suitable for structural applications, such as bearings, seals, armour, liners, nozzles and cutting tools. Thirdly, the thermally and chemically stable ceramic composites could revise their high thermal conductivity and be suitable for high temperature components such as in jet engine and brake disks for aircrafts [102]. Further, CNTs/GNPs can also convert ceramics into functional materials for aerospace and automobile industries, such as knock sensors, seat pressure sensors, temperature sensors, oil sensors, impact sensors and road surface sensors, whilst the outstanding electrical properties of CNTs/GNPs make Al_2_O_3_ ceramic attractive for specific applications like heating elements, electrical igniters, electromagnetic/antistatic shielding of electronic components, electrode for fuel cells, crucibles for vacuum induction furnaces and electrical feed through [44,74,86,103,104,105]. Table 3 summarises the potential industries may have benefits from ceramic nanocomposites reinforced with CNTs and graphene. As the research is progressing in this important area, novel CNTs-reinforced ceramics with stunning properties are expected and may substituted several automobile and aerospace components in future furthermore, owning functionalities these have potential for third generation nanodevices.

**Table 3:** Potential application of key ceramics nanocomposites reinforced with CNTs and graphene.

References	Ceramic matrix	Reinforcing agent	Key properties	Parts/Components	Potential industries
[101]	Al_2_O_3_	CNTs/graphene	Wear resistance, high toughness, electrical properties, thermal properties	Cutting tools, corrosion/erosion resistance pipes, electrical contacts, armour plates	Automobile, petrochemical industry, electric component manufacturing, defence industry
[106]	Si_3_N_4_	CNTs/graphene	Excellent mechanical, chemical, and thermal properties	Gas turbines, aircraft engine components and bearings	Power generation, aerospace, automobile sector
[107]	BaTiO_3_	CNTs/graphene	Ferroelectrics, piezoelectric and colossal magnetoresistor properties	Electric generator, computer hard disks, sensors	Renewable energy, power generation, electronic, computer manufacturing, data storage, aerospace industry
[108,109,110]	ZrO_2_	CNTs/graphene	High mechanical properties, excellent fracture toughness, elevated temperature stability, high breakdown electrical field and large energy bandgap	Solid oxide fuel cells, oxygen sensors and ceramic membranes	Renewable energy, chemical industry, water desalination sectors
[111,112,113]	TiN and FeN	CNTs/graphene	Excellent electrical properties	Capacitors, electronic conductor in electronic devices	Electrochemical industry, power and electronic sector, aerospace and automobile industries
[114]	Mulite	CNTs/graphene	High in electric and optical properties	Sensor	Electronic industry, aerospace sector and automobile industry

## 5. Conclusions

Advanced in the ceramics reinforced with carbon nanostructures (CNTs and graphene) have been thoroughly reviewed. Successes in the purification and dispersions of MWCNTs are somehow satisfactory, however SWCNTs need further research and standards for CNT dispersion are vital for addressing the quality and reliability with confidence. Microwave sintering has potential for producing dense nanocomposites and may eliminate the CNT damage problem associated with the hot-pressing, and by adopting to standard testing methods fracture toughness discrepancies could be reduced. CNTs-reinforced ceramics follow the combined advanced toughening mechanisms of CNT’s stretching/uncoiling and the classical fibre pullout theory, as an energy dissipating process. Rough surface and nanopits of MWCNTs explain the strong interface connections with ceramic matrix and the confirmation of the formation of intermediate Al_2_OC or Al_4_C_3_ phases at the interface further strengthens these explanations. Conclusively, problems of reinforcing MWCNTs into ceramics have been solved to some extend; however, the addition of SWCNTs still carries questions. Despite challenges and controversial issues, CNTs have successfully enhanced the toughness and other properties of brittle ceramics and converted them into useful materials for next generation applications.

It is clear that graphene can play an important role as filler in ceramics according to publications. In addition to the exceptional mechanical properties of GNPs which are similar to CNTs, researches have shown that GNPs can be more easily dispersed in ceramic matrix than CNTs which is the key challenge in preparing ceramic composites. Additionally its 2D and flexible microstructure introduced a new toughening mechanism in the ceramic matrix (anchoring around the grain) that could significantly absorb energy against crack propagation and delay the fracture. However, work on graphene ceramic composites is in its early stages and there are still considerable works that need to be done in order to optimise their processing, microstructure and interfacial properties to obtain better multifunctional properties from graphene-ceramic composites.
